# NeuraEngineDx: An IoT condition monitoring device for Small-Scale fishing vessel engines

**DOI:** 10.1016/j.ohx.2026.e00763

**Published:** 2026-03-21

**Authors:** Yuniar Endri Priharanto, Indra Jaya, Ayi Rahmat, Medria Kusuma Dewi Hardhienata, Donwill Panggabean

**Affiliations:** a Department of Marine Science and Technology, IPB University, Bogor, Indonesia; b School of Data Science, Mathematics and Informatics, IPB University, Bogor, Indonesia; c Department of Postgraduate School, Universitas Terbuka, Jakarta, Indonesia

**Keywords:** NeuraEngineDx, Engine Monitoring, Condition Maintenance, Small-scale Fisheries, Multi-sensors

## Abstract

NeuraEngineDx is a multi-sensor device to monitor condition of small-scale fishing vessel engines and is intended for IoT-based condition maintenance applications. The system integrates temperature sensor, vibration, sound and an infrared sensor, with an RTC DS3231 module for precise time-stamping. Data are collected in real time, logged to a MicroSD card, and can be transmitted to a cloud server via internet connectivity for remote analysis and condition monitoring. The enclosure is 3D-printed using Nylon Carbon (PA6-CF) to withstand high temperatures and harsh field conditions, while the circuit board is manufactured using FR4 material to ensure electrical stability. Performance validation against reference instruments demonstrated high metrological integrity, particularly for temperature and rotational speed, with R^2^ values of 0.997 and 0.989, respectively. Quantitative metrics revealed a Mean Absolute Percentage Error (MAPE) of 1.11% for temperature and 0.36% for RPM, with a relative expanded uncertainty (Rel. U) below 1% for both parameters. While the sound and vibration sensors exhibited higher variances due to stochastic ambient noise, the system effectively identifies engine parameter deviations under simulated fault conditions. Limitations include susceptibility of the sound sensor to ambient noise, dependency on internet signal quality for cloud data transmission, and increased power consumption during extended modem activity. NeuraEngineDx provides an efficient, robust, and flexible platform for research and condition monitoring applications, particularly for small-scale diesel engines used by artisanal fisheries.

## Graphical abstract

**Figure:**
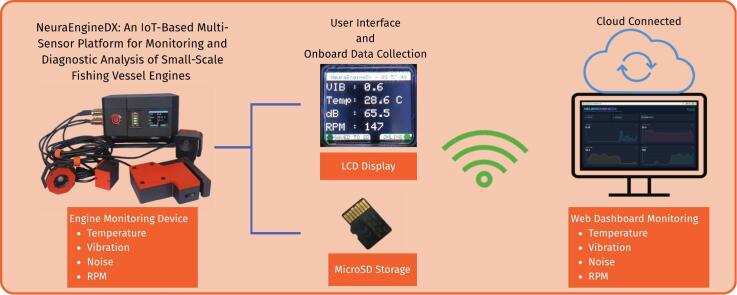

**Specifications table:** 

Hardware name	*NeuraEngineDx*
Subject area	• Engineering • Open source alternatives to existing infrastructure • General
Hardware type	• Field measurements and sensors
Closest commercial analog	*The Vib360 Engine Condition Monitoring System (See*Table 1*for a detailed quantitative comparison of cost, features, and accuracy).*
Open source license	*Creative Commons Attribution-ShareAlike 4.0 International License (CC BY-4.0)*
Cost of hardware	*USD 144.88 (A Detailed cost breakdown is presented in*Section 4*, Bill of Materials)*
Source file repository	https://doi.org/10.17605/OSF.IO/U43ZF (https://osf.io/u43zf/overview)

## 1 Hardware in context

In the maritime and fisheries sector, particularly within small-scale fisheries, the reliability of marine propulsion engines is a critical factor that directly influences both operational safety and the sustainability of fishing activities [1], [2], [3]. An unpredictable engine failure at sea not only results in economic losses due to reduced operational time but also poses a significant safety risk for fishers during fishing operations [4], [5]. In general, small-scale fishing vessels with a length of less than 12 m are not equipped with quantitative engine condition monitoring systems, resulting in operations that rely heavily on the operator’s experience and subjective judgment [6]. These vessels operate under one-day fishing scheme, with operational durations ranging from 15 h per trip depending on the target species and gear [7], [8]. During active fishing seasons, vessels often operate on a daily or near-daily basis, resulting in prolonged engine usage under varying load conditions, which fluctuate significantly between high-output steaming and low-load fishing or drifting phases [7], [9].

Several accident investigations and maritime safety studies indicate that machinery and engine failure represent a dominant cause of incidents in fishing and commercial vessels. An analysis of the UK Marine Accident Investigation Branch (MAIB) database identified 233 accidents related to machinery damage, accounting for 62.97% of the investigated cases in that study [6], [10]. On a broader scale, machinery failure is estimated to contribute to approximately 25% of global maritime incidents [11], while regional analyses report comparable figures, including 35% of reported accidents in the Aegean Sea between 1999 and 2009 being attributed to hull or machinery failure [12]. In small-scale fishing operations, machinery-related casualties are particularly critical; a study of Peruvian fishing vessels recorded 564 casualties associated with machinery damage and grounding, with engine failure being a major contributor to vessel drifting emergencies [13]. Insurance and failure statistics further reinforce this trend, showing that 28% of machinery-related insurance claims are caused by main engine failures [14], and that approximately 30% of engine room failures originate from engine-related problems [15]. At the component level, diesel engine injection systems account for approximately 27–28% of documented engine failures, highlighting the importance of early detection of mechanical degradation [16]. Addressing these machinery-related risks requires a clear understanding of the common failure modes in marine diesel engines and their corresponding signatures observable through physical signal analysis.

In the context of marine diesel engines, common engine malfunctions include cylinder combustion imbalance, bearing wear, valve clearance abnormalities, and cooling system failures [14], [17]. For instance, combustion imbalance manifests as increased torsional vibration and non-uniform angular velocity [18], [19]. Bearing degradation is characterized by high-frequency energy peaks in the vibration spectrum [20], [21]. Furthermore, cooling system failures, such as pump malfunctions or coolant blockages, result in a rapid and sustained rise in the thermal profiles of the cylinder head and lubricant paths [9], [14]. Valve abnormalities typically generate distinct impulsive knocking sounds [17]. Identifying these characteristics is critical for early diagnosis; therefore, the integration of vibration, temperature, and acoustic sensors in a Condition Monitoring System (CMS) enables a multidimensional assessment of these mechanical health indicators, ensuring that subtle deviations are detected before they lead to catastrophic engine failure [18], [22], [23].

In practice, the adoption of CMS in small-scale fisheries remains limited, primarily due to the high cost and complexity of industrial grade systems [24]. However, recent advancements in sensing technology, the Internet of Things (IoT) [25], and cloud computing [26], [27], [28] have enabled the development of open-hardware platforms and the implementation of CMS that are low-cost, portable, energy-efficient, and fully open-source [25], [26], [28], [29]. Its implementation has also demonstrated strong reliability in the energy sector, particularly in wind turbine condition monitoring [21], [30], as well as for fault detection and predictive maintenance in photovoltaic power plants [31]. In the mining industry, IoT technologies are employed to monitor performance and optimize heavy equipment operations, including haul trucks and excavators, under harsh environmental conditions [32]. Furthermore, IoT has advanced healthcare through wearable sensor based remote monitoring using low power protocols and mesh network wireless nurse call systems that improve response time and patient safety while simplifying hospital infrastructure. [33], [34]. Comparable efficiency gains are observed in the food production sector, where the integration of smart sensors and artificial intelligence techniques, including deep learning, facilitates real time quality monitoring, significantly reduces production waste, and enhances profitability without imposing excessive operational costs [35], [36].

Building on these cross-sector advancements, predictive maintenance strategies have been increasingly implemented across various industrial sectors to provide real-time information on the operational status of mechanical equipment [37], [38], [39]. As an industrial benchmark, commercial systems such as Vib360 are designed to provide real-time visibility of engine health through torsional vibration monitoring. These systems employ non-intrusive sensors to detect mechanical deviations that serve as indicators of common engine faults, including cylinder combustion imbalance, bearing wear, and shaft misalignment. A comparative overview of the proposed open-hardware pllatform and a representative commercial solution is presented in Table 1.

**Table 1:** Comparison between NeuraEngineDx and Commercial Benchmark (Vib360).

**Feature**	**NeuraEngineDx (proposed)**	**Vib360 (commercial)**
Sensor Configuration	Multi-sensor system (temperature, vibration, acoustic, and RPM)	Torsional vibration and peak pressure analysis
Sensor Architecture	Integrated multi-modal sensing unit	Dual non-intrusive sensor architecture
Initialization Method	Instant baseline establishment without historical data	Instant baseline establishment without historical data
System Flexibility	*Open-source and modular design (ESP32-based)*	Retrofittable system compatible with multiple engine models
Connectivity and Monitoring	Autonomous IoT node with cloud synchronization	In-situ monitoring and remote fleet management
Accessibility	Optimized for small-scale vessels	Primarily designed for industrial and large-scale vessels
Unit Cost	USD 144.88 (BOM-verified)	Quotation-based (Industrial-tier pricing)

These indicators require continuous multi-parameter monitoring to support predictive maintenance. However, commercially available systems capable of capturing such signals remain economically inaccessible for small-scale fisheries, reinforcing the need for an affordable and context-specific alternative. To address this gap, the proposed system integrates commonly used low-cost sensors, including vibration, temperature, rotational speed, and acoustic sensors, each contributing complementary information on engine condition. Unlike existing condition monitoring platforms that are primarily designed for industrial environments, the proposed system introduces a maritime-resilient architecture specifically engineered for small-scale fishing vessels. The core novelty lies not merely in multi-sensor integration, but in the hybrid connectivity framework that ensures data continuity in blind-signal maritime zones, combined with an autonomous dual-layer storage strategy and a thermally resistant Nylon Carbon enclosure. This integrated design directly addresses the operational, environmental, and connectivity constraints unique to small-scale fisheries.

In this study, NeuraEngineDx was implemented using an MLX90614 infrared temperature sensor, a MEMS-based MPU-6050 accelerometer for vibration measurement, an infrared sensor for engine rotational speed measurement, and a MAX4466 microphone-based sound sensor. The device is portable, and compatible with the constraints of small fishing vessels, supporting enhanced onboard safety and condition awareness.

The contributions of this study can be summarized as follows:

1. Design and implement a multi-sensor monitoring system for small-scale fishing vessel engines.
2. Integrates temperature, vibration, and sound sensors to provide a comprehensive indicator of engine operating conditions.
3. Provides a portable and real-time instrumentation platform that mitigates operational failures by advising on developing predictive maintenance requirements, identified through continuous monitoring of engine performance indicators
4. Establishes a foundation for the development of an IoT-based CMS tailored to small-scale capture fisheries operations.

## 2 Hardware description

The developed hardware is designed as an integrated and user-friendly engine monitoring system, particularly suited for small-scale fishing vessels. It enables real-time monitoring of engine conditions across a wide range of operating environments, from controlled laboratory tests to dynamic marine settings. The system can be customized directly in the field, allowing flexible sensor configuration and ensuring reliable data acquisition under real operating conditions. This adaptability makes the platform valuable not only for practical use by small-scale fishermen, but also for academic research, model development, and multidisciplinary experimental studies.

The primary advantages of this system lie in its relatively low cost, ease of operation, and the use of readily available sensor modules that are inexpensive to replace when needed. These characteristics make it a practical alternative to industrial monitoring devices, which are often significantly more expensive and difficult to modify. With its open interface and IoT-based integration capabilities, the hardware is well-suited not only for real-time machinery condition monitoring but also as a research development platform that supports the exploration of new designs in the fields of marine engineering, fisheries, safety, condition monitoring, fault diagnosis, and predictive maintenance.

### 2.1 System architecture and operational framework

**Fig. 1:**
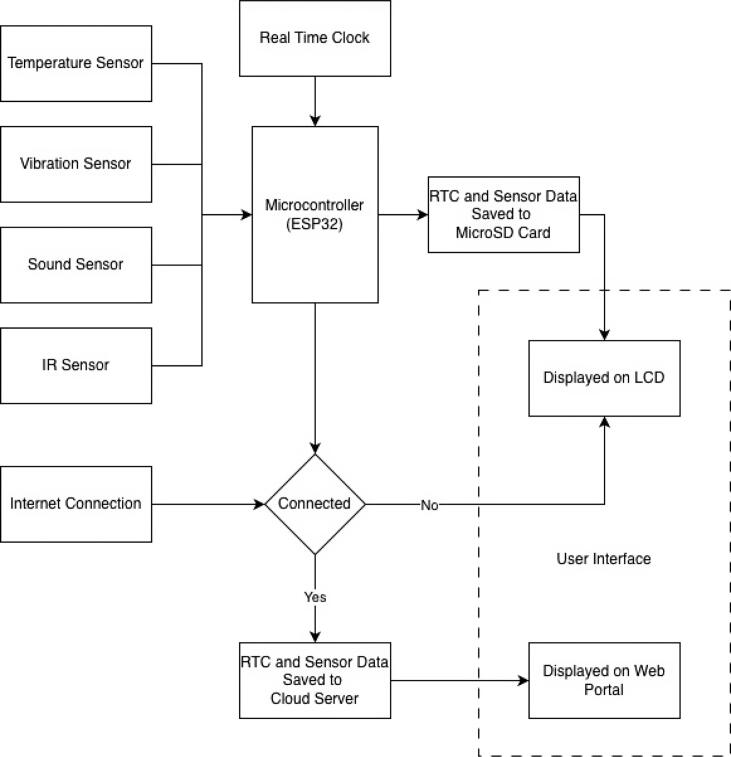
Operational framework of the NeuraEngineDx.

The operational framework of the NeuraEngineDx system is illustrated in Fig. 1. The overall architecture is divided into three main stages: data acquisition, local processing and storage, and conditional data transmission.

In the first stage, the ESP32 microcontroller acquires signals simultaneously from four primary sensors measuring temperature, vibration, sound level, and rotational speed. In the second stage, all acquired data are time-synchronized using a real-time clock (RTC) module and automatically stored locally on a MicroSD card in CSV format to ensure data integrity. The third stage involves real-time visualization and conditional data transmission to a remote server.

The system is specifically designed for marine environments with limited or intermittent network connectivity. When an internet connection is unavailable, real-time sensor readings are displayed locally on a 1.8″ TFT LCD embedded in the device, enabling fishermen and operators to continuously monitor engine conditions while operating offshore. By observing these real-time data, operators can identify deviations from the normal operating baseline of the engine. For example, sudden increases in temperature may indicate potential cooling system failures, while elevated vibration levels may suggest mechanical issues such as bearing wear or shaft misalignment. Once internet connectivity becomes available such as near coastal areas, islands, or ports, previously stored data are synchronized with a cloud server and made accessible through a web-based dashboard. This hybrid monitoring approach, combining local visualization and remote data access, enables data-driven assessment of engine condition and provides actionable information that supports proactive maintenance before critical failures occur.

### 2.2 Communication and network configuration

In the proposed network architecture, NeuraEngineDx employs an ESP32 microcontroller for data acquisition, local processing, and communication. The ESP32 connects via Wi-Fi to a portable 4G cellular modem configured as a wireless access point. The modem provides internet backhaul through a 4G mobile network, enabling remote data transmission without dependence on fixed onshore infrastructure. This hybrid Wi-Fi–cellular configuration allows NeuraEngineDx to function as an autonomous IoT node in coastal or remote fisheries environments where wired Wi-Fi infrastructure is not available. System operation is limited only by cellular network coverage.

Data communication between the device and the cloud server is implemented using an HTTP-based RESTful architecture. Sensor data are transmitted periodically via HTTP POST requests. On the server side, a lightweight PHP-based backend processes incoming data, validates payloads, and stores them in a MySQL relational database for visualization and further analysis through a web-based dashboard.

### 2.3 Data acquisition and sampling configuration

NeuraEngineDx employs a hybrid time-based and event-driven data acquisition approach, considering microcontroller processing limitations and communication stability. This prevents I2C bus congestion during simultaneous multi-sensor polling. Instead of a fixed global sampling rate, the configuration is tailored to the physical characteristics of each measured signal.

The MLX90614 infrared temperature sensor is sampled at an effective interval of 1 s, which is sufficient to capture the thermal dynamics of marine engines. This sensor was selected over contact-based alternatives due to its non-intrusive measurement capability and adequate ± 0.5°C accuracy. Engine rotational speed is measured using an infrared pulse-based sensor, with RPM values calculated every 1 s based on the number of detected pulses within the sampling interval.

Vibration measurements in this system are acquired using the MPU6050 accelerometer through a burst sampling approach, in which a sequence of acceleration samples expressed in gravitational units (g) is collected over a short time window. This sensor was selected by the need for a low-profile, integrated 3-axis solution that allows for digital signal processing (DSP) within the I2C bus. The acquired acceleration data are band-limited using a 21 Hz low-pass filter to suppress high-frequency noise and drift and subsequently reduced to a representative peak value. To comply with common condition monitoring practices for rotating machinery, the filtered acceleration signals are converted into vibration velocity expressed in mm/s through numerical integration in the time domain. Vibration velocity is selected as the primary indicator because it provides a more reliable representation of mechanical fault severity in low- to medium-speed engines commonly used in small-scale fishing vessels, while avoiding the need for continuous high-frequency data acquisition.

Sound level measurements are obtained using the MAX4466 microphone sensor with a time-window sampling method. Analog audio signals are acquired over a 50 ms window to calculate peak-to-peak amplitude. Sound intensity is then expressed in decibel (dB) units using an inferential approach.

All sensor data are logged locally on the MicroSD card at 1-second intervals. Each data record includes vibration peak value, object temperature, sound level, and engine speed, along with a timestamp provided by the RTC module. This local storage strategy ensures zero data loss during periods without internet connectivity.

For cloud transmission, locally stored data are accumulated over 10 s intervals, processed to compute average values for each sensor parameter, and transmitted as a single data packet. This strategy reduces communication load, conserves bandwidth, and improves data transmission reliability in environments with unstable network quality.

### 2.4 Hardware components and sensor specifications

The main components of the NeuraEngineDx system include:

1. ESP32 TTGO board serves as the main processing unit, featuring a dual-core 240 MHz processor and 520 KB of SRAM memory.
2. The sensor assembly, consisting of:
  a. MLX90614 (Non-contact infrared temperature sensor).
  b. GY-521 (Vibration sensor based on the MPU6050 accelerometer).
  c. MAX4466 (Microphone sound sensor).
  d. IR Sensor – Optical sensor for measuring rotational speed (RPM).
3. The power supply consists of a 5 V DC system, including:
  a. Nine 18,650 Lithium-ion Batteries, configured in 3-series 3-parallel (3S3P) arrangement, providing 12 V and 9000 mAh.
  b. Battery Management System (BMS), 3S 12.6 V, 20 A for overcharge, over-discharge, and overcurrent protection.
  c. XL4015 DC–DC Buck Converter (Step-Down), Input Voltage: 5–38 V DC, Adjustable Output Voltage: 1.25–36 V DC.
  d. USB Type-C Charging Module, Input Voltage: 3–6 V DC, Output Charging Voltage: 16.8 V DC.
  e. 3S 12.6 V Lithium Battery Capacity Indicator.
  f. Push Button Switch, Rated for DC 3–24 V.
4. MicroSD card (Class 10, ≥ 4 GB) for data acquisition and system log storage.
5. Real-Time Clock (RTC DS3231) module for precise timestamping of measurements.
6. 1.8″ TFT LCD SPI display (128 × 160 pixels) for real-time visualization of measurement results.
7. Nylon Carbon (PA6-CF) enclosure, resistant to high temperatures and suitable for applications requiring direct contact with engine.
8. Portable 4G Wi-Fi modem, functioning as an internet gateway for cloud connectivity.

Detailed sensor specifications are provided in Table 2.

**Table 2:** Sensors Specification.

**Sensor**	**Measured Parameter**	**Specifications**
MLX90614	Temperature	Accuracy: ±0.5°C; Resolution: 0.02°C; Measurement range: −70°C to + 380°C; Interface: I2C
GY-521 (MPU6050)	Vibration	Resolution: 16-bit ADC; Acceleration range: ±2g, ±4g, ±8g, ±16 g; Interface: I2C
MAX4466	Sound Level	Frequency range: 20 Hz to 20 kHz; Adjustable gain: 25 × to 125×; Interface: Analog
IR Speed Sensor	Rotational Speed (RPM)	Type: Slot-type optocoupler; Operating voltage: 3.3 V to 5 V; Detection Distance: 2 ∼ 30 cm Output: Digital (pulse)

### 2.5 Power consumption

NeuraEngineDx is powered by an integrated lithium-ion battery pack with a total capacity of 9000 mAh, configured to support long-duration operation in marine environments. This capacity is designed to ensure continuous monitoring throughout typical small-scale fishing activities, including one-day fishing operations.

A power consumption analysis was conducted based on typical operating conditions, distinguishing between offline operation (local data acquisition and display only) and online operation (local operation combined with wireless data transmission). Table 3 summarizes the estimated current consumption of each system component under both operating modes.

**Table 3:** Power consumption analysis of NeuraEngineDx.

**Component**	**Working Offline (mA)**	**Working Online (mA)**
ESP32 TTGO T-Call v1.4	∼100	∼180
MLX90614 Temperature Sensor	∼2	∼2
MAX4466 Sound Sensor	∼1	∼1
GY-521 (MPU6050) Vibration Sensor	∼5	∼5
IR Sensor	∼2	∼2
1.8″ TFT Display (ON)	∼50	∼50
Battery indicator (LED)	∼2	∼2
Power button (LED)	∼2	∼2
Buck converter + losses	∼20	∼20
Total Current Consumption	∼184 mA	∼264 mA
Battery Capacity	9000 mAh (11.1 V – 12.6 V Li-ion)	
Estimated System Power	∼2.04 W	∼2.93 W
Estimated Operating Duration	∼48 h	∼34 h

When operating offline, the system draws an average current of approximately 184 mA, resulting in an estimated continuous operating duration of up to 48 h per full battery charge. In online operation, where wireless communication via the 4G gateway is active, the average current consumption increases to approximately 264 mA, yielding an estimated operational duration of approximately 34 h. This duration significantly exceeds the typical operational time of small-scale fishing vessels, which generally ranges from 8 to 12 h per trip, thereby ensuring uninterrupted engine condition monitoring throughout daily fishing activities.

The increase in power consumption during online operation is primarily attributed to the higher current demand of the ESP32 microcontroller during active data transmission. Despite this increase, the system maintains sufficient battery endurance to support extended monitoring without the need for mid-operation recharging. System power status can be monitored in real time through the integrated battery capacity indicator, allowing operators to assess remaining energy during operation and ensuring reliable system performance while at sea.

### 2.6 System integration and practical Considerations

**Fig. 2:**
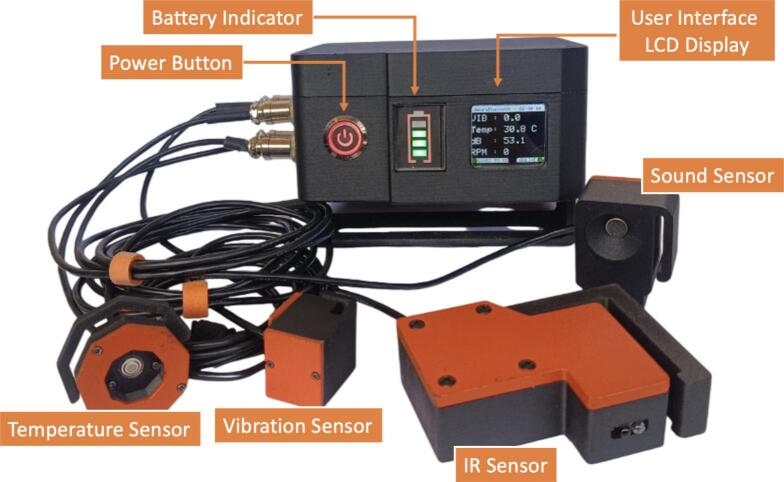
NeuraEngineDx, hardware to monitor small-scale fishing vessel engines.

**Fig. 3:**
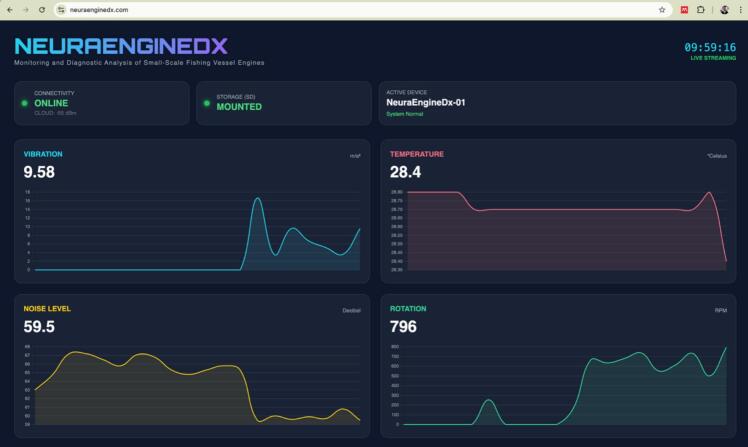
Web Based dashboard for monitoring engine parameter.

All sensors are connected to ESP32 through I^2^C buses or analog inputs pins. Acquired data are automatically stored in CSV format on a microSD card and displayed locally on the LCD screen. The complete hardware configuration is shown in Fig. 2. while the web-based dashboard is presented in Fig. 3.

The sensor configuration within each sensor case was designed to allow the replacement of a single sensor without disrupting the operation of other sensors. This modular design enables continuous monitoring even if one sensor malfunctions or fails, facilitating rapid maintenance and repair.

In addition, NeuraEngineDx is designed as a portable and non-intrusive monitoring instrument that does not require permanent installation on the engine. The device can be easily mounted and removed using a simple mounting mechanism, making it adaptable to various small-scale fishing vessels and allowing safe removal when the vessel is not in operation. The maintenance cost of the system is relatively low, as it utilizes widely available components and modules that are affordable for small-scale fishermen. The operational cost of NeuraEngineDx is also minimal because it is powered by rechargeable batteries, which can be recharged using standard USB Type-C adapters commonly used for mobile devices, eliminating the need for specialized power adapters.

NeuraEngineDx is designed as a monitoring instrument and does not replace standard preventive maintenance procedures such as lubricant replacement, filter changes, or routine mechanical inspections. Instead, it serves as an early fault identification tool, enabling fishermen to detect abnormal operating conditions outside regular maintenance schedules and to perform proactive interventions before severe engine failure occurs.

Some of the direct benefits for fishermen include the following:

1. The system can identify early signs of engine malfunction, such as excessive vibration, elevated temperature, or abnormal noise, enabling operators to perform predictive maintenance before severe damage occurs.
2. Reducing operational costs. By enabling early detection of equipment failures, fishermen can avoid incurring substantial repair expenses and minimize downtime caused by engine malfunctions during operations.
3. Enhancing at sea work safety. A vessel breakdown at sea can pose significant hazards. Real-time monitoring enables early detection of potential failures, thereby reducing the risk of sudden malfunctions and ensuring the safety of crew members.
4. User-friendly operation without advanced technical expertise. The system is designed with simplicity in mind, providing clear data displays and intuitive indicators. This allows fishermen to understand the engine’s condition without requiring specialized technical knowledge.
5. Improving maintenance efficiency. Fishermen can schedule maintenance based on the actual condition of the engine rather than relying on estimated time intervals. This condition-based approach complements preventative maintenance practices, helping to optimize maintenance operations and avoid unnecessary labor and costs.

This hardware platform also offers several benefits for researchers, academics, and technicians by providing valuable advantages for studies in the marine and fisheries sector, particularly in small-scale fishing vessel engines, maintenance strategies, and condition monitoring, as follows:

1. Sensor flexibility. Enables the use of various sensor combinations (vibration, noise, temperature, RPM) configured to specific research objectives.
2. Cost-effectiveness. Offers an affordable alternative for field research.
3. Ease of IoT integration. Supports real-time data acquisition and transmission, facilitating cloud-based or big data experiments.
4. Open research platform. Serves as a foundation for developing new designs or algorithms in machine monitoring, fisheries, and maritime applications.

The NeuraEngineDx is designed not only to support scientific research but also to provide tangible benefits for small-scale fishers who rely daily on boat engines as their primary source of propulsion. Compared with existing industrial monitoring systems, which are often expensive and complex, this device offers a simpler, cost-effective, and user-friendly solution that is suitable for field operators.

## 3 Design files summary

The CAD models for the 3D-printed enclosure were created using Onshape 1.182 and subsequently exported in STL format for 3D printing. All CAD and STL files are provided alongside this article. The printing process was performed using PA6-CF filament on a Bambu Lab A1 printer, selected for its high mechanical strength and environmental durability.

The Printed Circuit Board (PCB) design was developed using EasyEDA, including the complete schematics and production files required for PCB fabrication. The entire mechanical design was realized in Onshape® and can be cloned or exported in multiple formats. The file structure is organized according to the component hierarchy and sub-assemblies, facilitating understanding, navigation, and potential modifications. Detailed files of the enclosure design, schematic design, PCB board, and firmware are summarized in Table 4.

**Table 4:** Design files summary.

**Design file name**	**File type**	**Open source license**	**Location of the file**
Enclosure Design			
1. Main Enclosure.stl	STL	CC BY 4.0	https://osf.io/pc8ar
2. Temperature Sensor Enclosure.stl	STL	CC BY 4.0	https://osf.io/ghr4c
3. Sound Sensor Enclosure.stl	STL	CC BY 4.0	https://osf.io/8vspu
4. IR Sensor Enclosure.stl	STL	CC BY 4.0	https://osf.io/4d28y
5. Vibration Sensor Enclosure.stl	STL	CC BY 4.0	https://osf.io/kz4nt
PCB Board			
1. Main Board.pdf	PDF	CC BY 4.0	https://osf.io/jmsp7
2. Extension Board.pdf	PDF	CC BY 4.0	https://osf.io/kudx6
3. MLX90614 Module Ext Board.pdf	PDF	CC BY 4.0	https://osf.io/9ckxg
4. MAX4466 Module Ext Board.pdf	PDF	CC BY 4.0	https://osf.io/jx2u8
5. IR Module Ext Board.pdf	PDF	CC BY 4.0	https://osf.io/h485p
6. GY-521 Module Ext Board.pdf	PDF	CC BY 4.0	https://osf.io/texz3
Schematic Design.pdf	PDF	CC BY 4.0	https://osf.io/6buap
NeuraEngineDx.ino	ino	CC BY 4.0	https://osf.io/c3fyn

Fabrication of the NeuraEngineDx requires basic electronics assembly skills (primarily through-hole soldering for headers and modular component mounting) and basic proficiency in 3D printing. The estimated total build time is approximately 40 – 48 h.

1. “Main Enclosure.stl” is the design for the main enclosure of the NeuraEngineDx, serving as a housing for all electronic components, the battery, and communication modules.
2. Sensor Enclosures (item 2 – 4) is a set of modular, task-specific housings designed to protect individual sensing modules from engine heat while ensuring signal integrity.
3. PCB Board folder is the printed layout file of the PCB, showing the routing of traces, component placement, and final board layout, ready for production.
4. “Schematic Design.pdf” is the complete electronic circuit schematic of the system, including the interconnections between sensors, communication modules, power supply, and the microcontroller.
5. “NeuraEngineDx.ino” is the firmware file for the device, containing all the programmed instructions required to operate the sensors, manage data processing, control communication functions, and run the user interface on the LCD display.

## 4 Bill of materials summary

The Bill of Materials detailing the components required to construct NeuraEngineDx is summarized in Table 5. The total cost for a single prototype is USD 144.88. The listed The listed prices are based on global marketplaces such as AliExpress and Amazon as of 2025. These costs are subject to regional sensitivity; for instance, local sourcing in regions with high import duties may increase costs by 15–20%, while bulk procurement of components could reduce the total BOM by approximately 25–30%. The use of off-the-shelf modules ensures that builders can find equivalent alternatives or local electronics distributors if specific vendors are unavailable.

**Table 5:** Bill of material summary.

**Designator**	**Component**	**Number of Unit**	**Cost Per Unit (USD)**	**Total Cost (USD)**	**Source of materials**	**Material type**
B1, B2,B3	18,650 Battery Pack (3S3P configuration)	1	26.48	26.48	Aliexpress	Li-ion
U2	BMS 3S 20A Protection Board	1	0.99	0.99	Aliexpress	Electronics
B4	XL4015 DC–DC Regulator Board	1	2.85	2.85	Aliexpress	Electronics
CN7, CN9	JST Connector − 2PIN	2	0.99	1.98	Aliexpress	Electronics
CN1, CN15, CN2, CN14	JST Connector − 3PIN	4	0.99	3.96	Aliexpress	Electronics
CN3, CN11, CN4, CN12, CN5, CN10	JST Connector − 4PIN	6	0.99	5.94	Aliexpress	Electronics
CN6, CN13	JST Connector − 6PIN	2	0.99	1.98	Aliexpress	Electronics
CN8	JST Connector − 8PIN	2	0.99	1.98	Aliexpress	Electronics
MC1	3S Type-C Charging Module	1	0.99	0.99	Aliexpress	Electronics
U1	ESP32 TTGO T-Call V1.4	1	16.51	16.51	Lilygo	Electronics
U8	DS3231 RTC I2C Module	1	1.63	1.63	Aliexpress	Electronics
U11	SPI MicroSD TF Card Reader Module	1	1.34	1.34	Aliexpress	Electronics
U14	16 mm Metal Push Button Switch (Self-Locking)	1	4.59	4.59	Aliexpress	Electronics
U15	Lithium Battery Level Indicator	1	1.8	1.8	Aliexpress	Electronics
U16	1.8″ TFT LCD SPI Display Module	1	8.54	8.54	Amazon	Electronics
U9	MLX90614 Infrared Temperature Sensor	1	11.99	11.99	Amazon	Electronics
U10	MPU6050 (GY-521) Accelerometer Module	1	1.89	1.89	Aliexpress	Electronics
U12	MAX4466 Microphone Sound Sensor	1	2.5	2.5	Aliexpress	Electronics
U13	IR Sensor Module	1	1.4	1.4	Aliexpress	Electronics
−	GX16-4 Pin Connector (H-24 Type)	4	2.67	10.68	Aliexpress	Electronics
−	25 mm Spacer Set (10 pcs)	1	5.99	5.99	Amazon	Brass
−	Male–Female Header Socket Set (40 Pin Header 2.54 mm)	1	2.89	2.89	Aliexpress	Electronics
−	Neodymium Magnet Block (20 × 10 × 2 mm, 20 pcs)	2	12.99	25.98	Amazon	Neodymium Magnet
−	Custom PCB Fabrication	1	14	14	Instagram JLCPCB	FR4
−	3D Printed Enclosure	1	84	84	Tokopedia JLC3DP	PA6-CF
	Total Cost of Materials (USD)			144.88		

The system comprises both electronic and structural components, selected based on reliability, availability, and suitability for field deployment. The power system employs 18,650 lithium-ion batteries in a 3S3P configuration, meaning three cells are connected in series and three such series strings are connected in parallel. This configuration provides a nominal voltage of approximately 11.1 V (3 × 3.7 V) with a total capacity three times that of a single cell, thereby delivering more stable power and extended operational duration. The battery management system is controlled by a 3S 20A BMS module, providing protection against overcharge, over-discharge, and overcurrent, while a XL4015 DC–DC regulator maintains voltage stability during operation.

The long-term operational cost is dominated by the battery subsystem. The 18,650 cells have an estimated lifespan of 300–500 cycles. Under typical one-day fishing operations, battery replacement is anticipated every 2 years, costing approximately USD 26.48. The Nylon Carbon (PA6-CF) enclosure is designed for high durability. However, periodic inspection of the infrared sensor’s optical window and vibration mounts is recommended to ensure sustained measurement accuracy and mechanical stability.

The main processing unit utilizes an ESP32 TTGO T-Call V1.4, which integrates Wi-Fi, GSM, and GPRS connectivity, enabling real-time remote data transmission.

The device enclosure was fabricated using 3D printing with Nylon Carbon (Polymaker PolyMide PA6-CF), selected for its high-temperature resistance, high tensile strength, low weight, and durability under extreme environmental conditions. The PCB was manufactured using FR4, a material known for its electrical stability and high thermal resistance across various operational conditions.

All materials are readily obtainable from local and international marketplaces, facilitating reproducibility and further development of the system by other researchers.

## 5 Build instructions

### 5.1 Fabrication of NeuraEngineDx main unit and sensor node enclosures

3D-print the files with the “.STL” extension (provided in the Enclosure Design section on Files Summary) according to their respective units. All components should be printed using PA6-CF or PA12-CF with a 50% infill and 0.15 mm layer height to ensure structural integrity and thermal resistance.

After printing, ensure that all components fit correctly. Some parts require reinforcement using M2 or M3 screws. If components do not fit properly, post-processing may be necessary depending on print quality, such as sanding surfaces, removing support structures and and adjusting the holes for screws, connectors, the SD card slot, the display, or the power button.

### 5.2 Assembling the main board and sensor connectors

#### 5.2.1 PCB design and Fabrication

The Main Board PCB was designed as a double-layer board to minimize crosstalk and maintain signal stability for analog sensors, whereas the Sensor Boards were implemented as single-layer PCBs.

The PCB consists of three main sections:

1. Main Board. The primary PCB contains header slots for the ESP32 TTGO and provides connections to the 5 V–3.3 V power rails, expansion pins for the power supply, sensor nodes, memory card, TFT LCD, and RTC module.
2. The Extension Board. Integrating the RTC DS3231, microSD module, and charging module, and provides JST XH2.54 connectors to establish a reliable connection between the Extension Board and the Main Board.
3. Sensor Board. Designed to accommodate the MLX90614, GY521, MAX4466, and IR sensors.

The PCB layout files (see design file summary) can be fabricated in-house or through professional manufacturing services such as JLCPCB, PCBWay, or similar providers. The recommended technical specifications are: FR-4 material with a thickness of 1.6 mm and a minimum trace spacing of 0.35 mm, as pre-configured in the PDF files. Professional PCB manufacturing is recommended for long-term deployment due to improved trace precision, solder mask quality, and tighter dimensional tolerances (±0.1 mm), particularly for densely routed layouts, as detailed in the supplementary materials (Appendix A, https://osf.io/dg4a5).

**Fig. 4:**
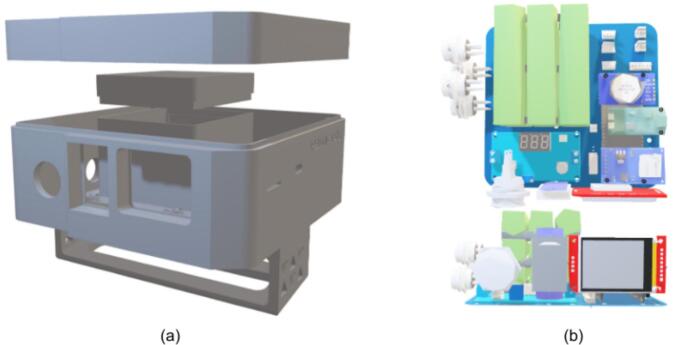
Design illustration of (a) the main enclosure and (b) the component layout within the main enclosure.

Fig. 4 presents the enclosure and internal layout, dimensioned to accommodate the finalized PCB design while providing mechanical protection, sufficient component clearance, and organized cable routing for marine deployment.

#### 5.2.2 Header pin and main component assembly

**Fig. 5:**
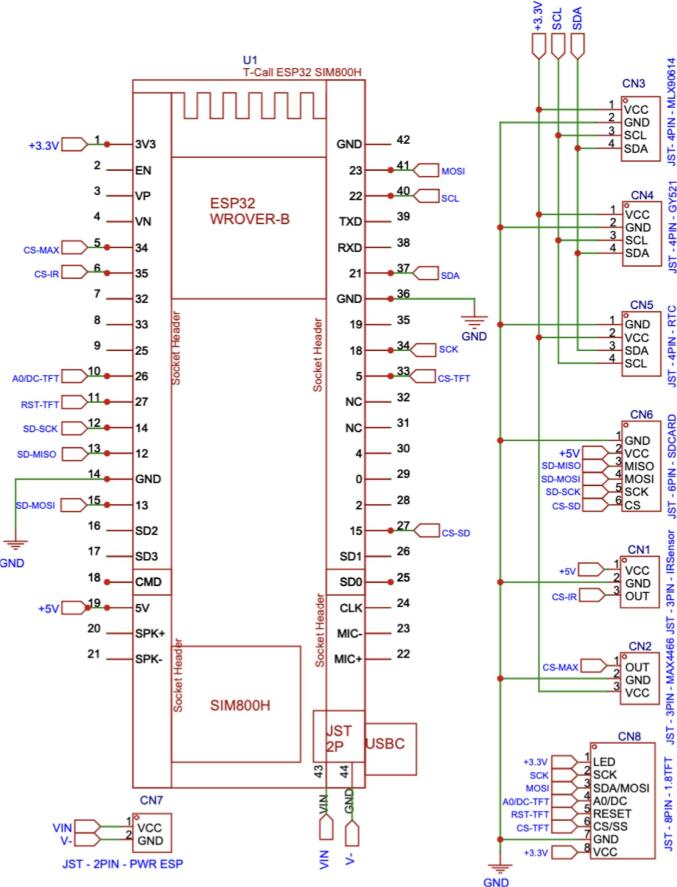
Schematic diagram of the main board, showing the ESP32 main processor and its connections to each module and sensor through JST XH2.54 connectors.

1. Install the ESP32 Header Pins and JST XH2.54 Connectors on the Main Board PCB
  a. Use a 2 × 21 male header and insert it into the solder holes on the ESP32 TTGO module. Proceed to solder all pins securely.
  b. Use a 2 × 21 female header (2.54 mm pitch) and insert it into the corresponding solder holes for the ESP32 TTGO on the PCB. Ensure the pin orientation matches the PCB design diagram, then solder all pins carefully.
  c. Install JST XH2.54 connectors as illustrated in the schematic design figure (Fig. 5). Multiple connectors need to be installed, ranging from 2-pin, 3-pin, 4-pin, 6-pin, up to 8-pin connectors, according to the PCB layout. Solder each connector at its designated position on the board as shown in Fig. 6.

**Fig. 6:**
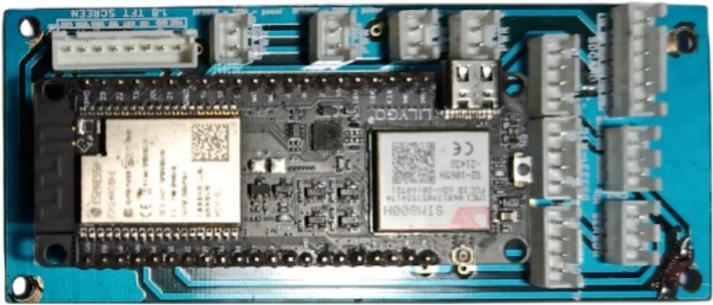
Main board with installed JST XH2.54 connectors and ESP32 module.
  d. Connect the LCD module to the mainboard via the JST XH2.54 cable connector and mount the LCD on the main monitoring unit.
  e. Inspect all solder joints to ensure there are no shorts, solder bridges, or cold joints.
2. Mounting Sensor Modules and Connectors on the Sensor Board
  a. Install the GX16-4 Pin Connector (H-24 Type) at the end of each sensor cable to allow easy detachment and reconnection to the Main Unit. Connect each cable end, both on the sensor side and the Main Unit side, according to its wiring path. The GX16-4 connector enables the Sensor Unit to be easily attached and detached.
  b. On the sensor side, attach male pin headers to the sensor module and solder them securely.
  c. Connect the soldered pin headers from the sensor module to the sensor PCB, then solder the headers and connect them to the corresponding sensor cables.
  d. On the Main Unit side, the GX16-4 connector is linked to the main board using a JST XH2.54 cable. Ensure that each sensor line is correctly assigned to its corresponding connector on the main board and verify that all cables are routed through the appropriate line.
  e. Mount the sensor PCB inside the sensor enclosure using screws, ensuring proper alignment and mechanical stability. Once positioned correctly, close and secure the enclosure cover.
  f. The sensor enclosure includes a designated space for neodymium magnets, allowing the enclosure to attach firmly to the monitored machinery.
  g. Fig. 7 illustrates an example of a vibration sensor connected to the GX16-4 connector.

**Fig. 7:**
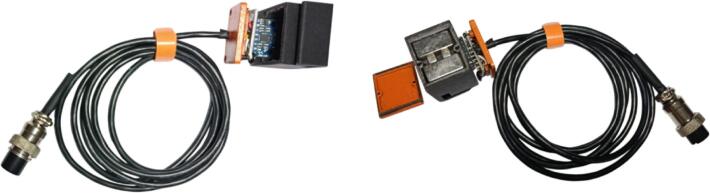
Sensor modules mounted within their respective enclosures, with cables and connectors linking each module to the main unit.
3. Installation of the microSD Module, DS3231 RTC, and Charging Module on the Extension Board
  a. For the microSD module, DS3231 RTC, and charging module, first install male pin headers and solder them securely.
  b. Connect the installed male pin headers to their designated positions on the extension board according to the circuit layout. Proceed by soldering the JST XH2.54 connectors, consisting of a 4-pin connector for the RTC module, a 6-pin connector for the microSD module, and a 2-pin connector for the charging module. Ensure that the JST XH2.54 cable routing from the main board aligns correctly with the corresponding traces on the extension board. A fully assembled extension board is shown in Fig. 8.

**Fig. 8:**
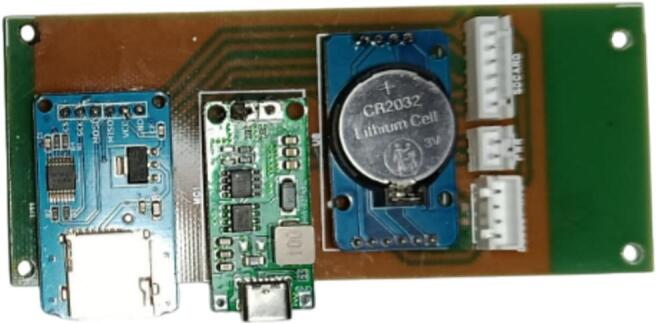
Installation of the microSD module, DS3231 RTC, and charging module on the extension board.
  c. Mount the extension board on top of the main board using spacers, ensuring that the microSD slot and charging module align precisely with their corresponding openings in the main monitoring unit enclosure.
4. Power Supply Installation
  a. Place the battery into the battery holder within the main monitoring unit, then close the battery holder cover and secure it using M2 screws.
  b. Connect the battery output to the input connector of the buck converter and solder the connection to the DC input terminal (5–12 V).
  c. Mount the XL4015 buck converter in its designated slot within the main monitoring unit, as shown in the schematic diagram.
  d. Connect the input of the buck converter to the power switch to enable controlled activation of the system.
  e. Connect the buck converter input in parallel with the battery level indicator to allow simultaneous voltage monitoring.
  f. Connect the buck converter input in parallel with the charging module to enable concurrent charging and operation.
  g. Connect the 5 V output from the buck converter to the onboard regulator of the ESP32 to supply stable voltage to the microcontroller.
  h. Install a lithium battery level indicator on the main power line to provide a visual indication of the battery’s charge level.

**Fig. 9:**
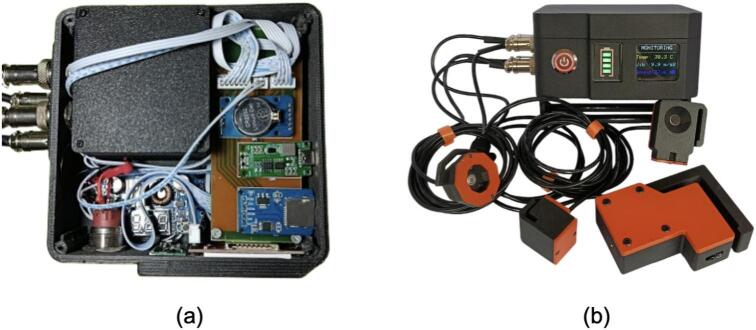
(a) Installation of the power supply, main board, extension boards, and sensor connectors on the main unit. (b) NeuraEngineDx Fully Assembled.

Fig. 9 presents the fully assembled NeuraEngineDx unit, integrating the enclosure, power system, main board, extension board, and sensor connectors in a compact configuration ready for testing and field deployment.

#### 5.2.3 Trace and connection testing

Prior to powering the system, a comprehensive verification of all connections should be performed:

1. Use a multimeter in continuity mode to check the connections between pins and power traces.
2. Ensure there are no short circuits between any traces in the circuit.
3. Perform a power supply test using a 5  V source and measure the voltage with a multimeter.
4. Connect the ESP32 board to a PC via USB to verify that the device is correctly recognized by the system.

Once all traces and connections have been successfully verified, proceed to the software configuration stage (Section 5.3).

#### 5.2.4 Potential risks and Mitigation

1. Wear protective goggles during soldering to prevent molten solder splashes.
2. Avoid prolonged soldering on the SIM800 module at a single point, as it is sensitive to heat.
3. Ensure the correct polarity of all connection before powering the system.
4. Apply all necessary safety precautions when performing soldering, wiring, cutting, or drilling operations.

### 5.3 Installing the software

Download and install the Arduino IDE.
Install the following required libraries: Wire.h, SPI.h, SD.h, Adafruit_MLX90614.h, Adafruit_Sensor.h, Adafruit_MPU6050.h, WiFiClientSecure.h, WiFi.h, HTTPClient.h, RTClib.h, TFT_eSPI.h, WiFiUdp.h, NTPClient.h, Adafruit_MLX90614.h.
Open the main program file (NeuraEngineDx.ino) provided in the Design File Summary.
Edit the Wi-Fi settings and adjust the username and password in the.ino file.
Rename the device_id in the.ino file by changing the number after “NeuraEngineDx-”. For example: NeuraEngineDx-02, NeuraEngineDx-03, and so on.
Upload the firmware to the ESP32 TTGO board using the Arduino IDE via a USB Type-C cable. A firmware troubleshooting guide is provided in the supplementary materials (Appendix B, https://osf.io/dg4a5).
Verify connectivity by ensuring that sensor data is displayed both on the LCD screen and on the cloud server (NeuraEngineDx.com).

## 6 Operation instructions

This section describes the operation of the NeuraEngineDx. The guidelines cover preparation procedures, standard operational protocols, data monitoring, and basic maintenance steps in field conditions.

### 6.1 Operational limits and Environment robustness

Before deployment, operators must adhere to the following technical limits to ensure metrological integrity and hardware longevity (Table 6).

**Table 6:** NeuraEngineDx Operational Limits.

**Parameter**	**Operational Limit**	**Mitigation Strategy**
Maximum Temperature	380°C (sensor), 180°C (enclosure)	Avoid direct contact with exhaust manifolds or other high-temperature surfaces.
Maximum RPM	2500 RPM	Limited by ESP32 interrupt processing latency and reflective marker integrity. Ensure stable marker attachment.
Vibration Tolerance	16 g	Secure mounting using integrated magnets and/or mechanical fasteners is mandatory.
Moisture Exposure	Non-submersible enclosure	Seal all ports when not in use and avoid prolonged exposure to moisture.
Water Resistance	Splash-resistant only	Avoid direct high-pressure washing or water jet exposure.
Corrosion Protection	No active corrosion coating	Periodically clean GX16-4 connectors using electrical contact cleaner.

### 6.2 Pre-Operation preparation

#### 6.2.1 Power and connection check

1) Ensure that the 18,650Li-ion batteries are fully charged (the indicator should show a minimum of 50% capacity).
2) Verify the voltage of the power cables before connecting the system to the main board (rechecking is not necessary if the main board has not been removed from its mounted position).
3) Confirm that all sensor connectors (I^2^C and analog) are securely attached and free from looseness.

#### 6.2.2 Storage Media check

1) Ensure that the microSD card is properly inserted into the SD slot.
2) Verify that at least 100 MB of free space is available for new data recording.

For a new card, the system will automatically create a.txt file upon the first run; ensure that the microSD card is formatted in FAT32.

#### 6.2.3 Communication connection

1) Ensure that the SIM card is correctly installed in the modem/router.
2) Confirm that cellular signal is available and stable.

#### 6.2.4 Sensor placement

**Fig. 10:**
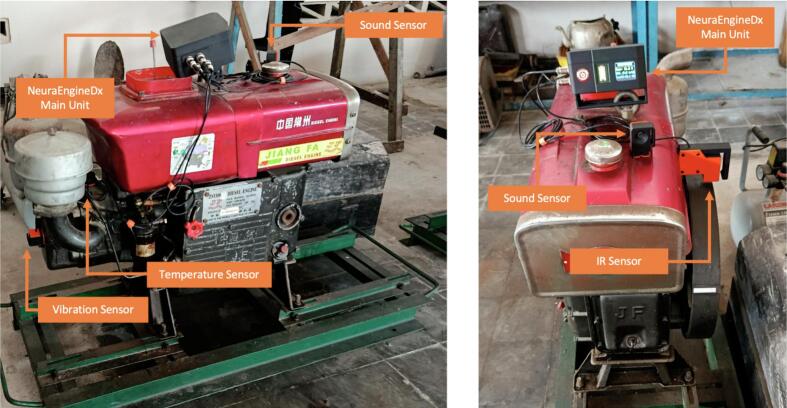
Placement of each sensor according to its operational position on the diesel engine.
2) Place each sensor according to its operational position:
  ▪ MLX90614: oriented toward the cylinder head to ensure direct thermal contact.
  ▪ GY-521: mounted on the surface of the cylinder head.
  ▪ MAX4466: positioned approximately 10–15 cm from the engine cylinder block.
  ▪ IR sensor: directed toward the flywheel with a reflective marker for accurate rotational detection.

1) Perform a visual inspection of the cables, connectors, and sensor mounting points. Verify the security of the main NeuraEngineDx unit, which should be positioned on top of the engine (Fig. 10). Ensure that the main unit does not obstruct coolant inlet or ventilation pathways.

### 6.3 System operation procedure

To operate NeuraEngineDx, the system must first be powered on and initialized to ensure proper functionality of all integrated sensors and communication modules. The procedure includes preparing the hardware for real-time data acquisition, verifying network connectivity, and ensuring safe operation during engine monitoring. The standard operating procedure is as follows:

1. Turn on NeuraEngineDx by pressing the main power button to initiate the internal booting process.
2. Wait approximately 10 s until the LCD screen displays a message indicating that the system is initializing and establishing an internet connection.
3. Once the connection is successfully established, the display switches to real-time data:
  ▪ VIB: xx
  ▪ Temp: xx
  ▪ dB: xx
  ▪ RPM: xx
4. Start the diesel engine and allow it to run at idle for a few seconds.
5. NeuraEngineDx automatically begins recording sensor data, including temperature, vibration, noise level, and engine RPM.
6. The LCD display updates the status of each sensor.
7. Sensor data are logged and stored on the internal MicroSD module.
8. Data are transmitted in real-time to the Cloud server.
9. A web-based dashboard visualizes engine parameters, such as cylinder head temperature, vibration level, sound intensity, and engine speed.
10. During operation, regularly inspect wiring paths to ensure they do not come into contact with moving components.

## 7 Validation and characterization

### 7.1 Sensor Calibration, Accuracy, and uncertainty

To ensure the accuracy and reliability of measurement data, all sensors integrated within the NeuraEngineDx system were calibrated prior to experimental testing on the engine. Calibration was performed by comparing sensor readings against industry-standard reference instruments under controlled conditions. This approach guarantees that the data used for monitoring purposes accurately represent the operational state of the engine.

1. Sensor Calibration

The MLX90614 infrared temperature sensor was used with its factory-calibrated settings. At the firmware level, temperature data are accessed via the I^2^C protocol using the readObjectTempC() function, which converts raw thermal readings into degrees Celsius (°C). A software-based validation filter is applied to remove anomalous measurements caused by signal interference or sensor faults. The data cleaning procedure is implemented as shown in Equation (1).

(1) $$T=\left\{\begin{array}{c} T_{\mathrm{raw}},\;\;\;\\ 0, \end{array}\;\right)\begin{array}{c} \mathrm{if}\;T_{\mathrm{raw}}\;⩽1000\;and\;T_{\mathrm{raw}}\;is\;not\;NaN\\ \mathrm{otherwise} \end{array}\;$$

Vibration measurements were performed using a three-axis MEMS accelerometer (MPU6050) configured with a ± 4 g measurement range and a 21  Hz bandwidth filter. To quantify vibration intensity in accordance with standard practices, the system calculates the total dynamic acceleration magnitude across the three axes (a_x_, a_y_, a_z_) while removing the static gravitational component (1 g ≈ 9.81  m/s^2^) to isolate mechanical vibrations. This computation is expressed in Equation (2), (3) [40], [41].

(2) $$a_{\mathrm{mag}}=\sqrt{a_{x}^{2}+a_{y}^{2}+a_{z}^{2}}$$

(3) $$V_{\mathrm{out}}=\left|a_{\mathrm{mag}}-9.81\right|\times C_{\mathrm{vib}}$$

Where $a_{\mathrm{mag}}$ represents the total acceleration magnitude (m/s^2^) and $C_{\mathrm{vib}}$ is a calibration constant. The constant $C_{\mathrm{vib}}$ is used to convert peak acceleration values into an estimated vibration velocity in mm/s, enabling direct comparison with reference vibration meters. To ensure data stability, the system applies a peak detection algorithm over 50 raw samples per data acquisition cycle to capture the maximum vibration intensity during engine operation.

The MAX4466 acoustic sensor produces an analog signal corresponding to sound amplitude. The analog voltage is converted into noise level using a peak-to-peak value approach. Calibration is performed both mechanically, by adjusting the sensor potentiometer, and in firmware by comparing sensor readings against a digital sound level meter reference. The peak-to-peak value is calculated according to Equation (4).

(4) $$V_{\mathrm{pp}}=V_{\max}-V_{\min}$$

Where $V_{\max}$ and $V_{\min}$ represents the maximum and minimum analog voltage values, respectively. The raw noise level is then expressed in decibels (dB) using a logarithmic relationship as shown in Equation (5) [42].

(5) $${\mathrm{Noise}}_{\mathrm{raw}}=20\log10\left(V_{\mathrm{pp}}\right)+C_{\mathrm{db}}$$

Where ${\mathrm{Noise}}_{\mathrm{raw}}$ denotes the sound pressure level in dB. The software applies an operational clamping range from 40  dB to 120  dB to ensure that the measurements remain within the human auditory range and the sensor’s sensitivity limits.

For RPM measurement using the IR sensor, the system employs a hardware interrupt in falling-edge mode to minimize latency and prevent pulse loss at high rotational speeds. A software debouncing mechanism is implemented to mitigate false pulses caused by signal reflections, with a minimum inter-pulse threshold of 3.000 μs, ensuring that only valid pulses are counted. RPM is determined by counting the number of pulses (*P*) within a 1,000  ms (1  s) time window, and the raw RPM value is calculated by multiplying the pulse count by a conversion factor to express rotations per minute, as shown in Equation (6).

(6) RPM=P×60

The measured RPM values are subsequently filtered using an exponential moving average (EMA) method to reduce fluctuations caused by noise and signal jitter.

2. Accuracy

To evaluate the performance of the NeuraEngineDx system, sensor measurements were compared against standard reference instruments. The agreement between the system readings and the reference data was quantified using four metrics: Coefficient of Determination (R^2^), Mean Absolute Error (MAE), Root Mean Square Error (RMSE), Mean Absolute Percentage Error (MAPE).

R^2^ assesses the strength of the linear relationship between NeuraEngineDx sensor readings and reference measurements. Values of R^2^ approaching 1 indicate a high degree of linearity. R^2^ is calculated as follows:

(7) $$R^{2}=1-\frac{\sum_{i=1}^{n}{\left(y_{i}-{\hat{y}}_{i}\right)}^{2}}{\sum_{i=1}^{n}{\left(y_{i}-\overset{¯}{y}\right)}^{2}}$$

Where $y_{i}$ is the actual value measured by the reference instrument, ${\hat{y}}_{i}$ is the value recorded by NeuraEngineDx, $\overset{¯}{y}$ is the mean of all reference measurements, and n is the total number of validation samples.

The Mean Absolute Error (MAE) quantifies the average absolute deviation between sensor and reference readings, ignoring the direction of the error.

(8) $$\mathrm{MAE}=\frac{1}{n}\sum_{i=1}^{n}\left|y_{i}-{\hat{y}}_{i}\right|$$

The Root Mean Square Error (RMSE) emphasizes larger errors, which is particularly relevant for detecting abrupt fluctuations in vibration and acoustic parameters.

(9) $$\mathrm{RMSE}=\sqrt{\frac{1}{n}\sum_{i=1}^{n}\left|y_{i}-{\hat{y}}_{i}\right|}$$

The Mean Absolute Percentage Error (MAPE) expresses the average error as a percentage of the reference value, providing a relative measure of accuracy independent of the unit of measurement.

(10) $$\mathrm{MAPE}=\frac{1}{n}\sum_{i=1}^{n}\left|\frac{{\hat{y}}_{i}-y_{i}}{{\hat{y}}_{i}}\right|\times 100\%$$

3. Uncertainty analysis

Measurement uncertainty was evaluated following the Guide to the Expression of Uncertainty in Measurement (GUM) framework. This analysis aimed to quantify the scientific doubt associated with each sensor parameter under the operational conditions of fishing vessel engines. The total uncertainty was calculated by combining two categories of error sources: Type A and Type B [43], [44], [45].

Type A Uncertainty (Repeatability).

Type A uncertainty was derived from the statistical variation of repeated measurements conducted under identical operating conditions. For each parameter, the standard deviation of the measurements (s) represented the uncertainty due to signal fluctuations and environmental noise. The standard Type A uncertainty was calculated using the following Equation (11).

(11) $$u_{A}=\frac{s}{\sqrt{n}}$$

where n is the number of samples.

Type B Uncertainty (Sensor Specification).

Type B uncertainty was obtained from the sensor accuracy specifications provided by the manufacturer. The maximum error limit was assumed to follow a uniform distribution, yielding the standard Type B uncertainty as:

(12) $$u_{B}=\frac{a}{\sqrt{3}}$$

Where a is the sensor error limit according to the datasheet.

Combined Uncertainty.

The combined standard uncertainty was computed using the root-sum-of-squares method:

(13) $$u_{C}=\sqrt{u_{A}^{2}+u_{B}^{2}}$$

To express uncertainty at an approximate 95% confidence level, a coverage factor k = 2 was applied to obtain the expanded uncertainty.

(14) $$U=2∙u_{C}$$

For inter-parameter comparison across different units, the relative uncertainty (Rel.U) was calculated as:

(15) $$\mathrm{Rel}.U\left(\%\right)=\left(\frac{U}{y_{\mathrm{ref}}}\right)\times 100\%$$

Where $y_{\mathrm{ref}}$ is the mean value of the reference instrument. The relative uncertainty provides a normalized metric to assess data acquisition quality relative to the measured signal, offering an objective evaluation of system performance across the operational range. The calculated uncertainty values ensure that deviations between sensor readings and reference measurements remain within acceptable measurement variation limits.

### 7.2 Validation and characterization

Validation and characterization were conducted to ensure that NeuraEngineDx operates according to its specifications and is capable of supporting scientific applications that require real-time machine condition data acquisition. The testing was performed through a series of experiments involving an infrared temperature sensor, a vibration sensor, a sound sensor, a rotational speed sensor, as well as data storage and communication modules, in order to evaluate the system’s response under various operational conditions.

The engine used in this study was a single-cylinder stationary diesel engine, representative of propulsion systems in small-scale fishing vessels. Its technical specifications are summarized in Table 7.

**Table 7:** Technical specifications of the diesel engine.

**Parameter**	**Specification**
Manufacturer / Model	Jiang Fa ZS1100
Engine Type	Horizontal diesel engine, four-stroke
Maximum Power Output	16 HP
Rated Rotational Speed	2200 RPM
Net Weight	150 kg

The system was tested by performing simultaneous multi-sensor data acquisition, recording data to a MicroSD card, displaying information on a TFT module, and transmitting data via a cellular network using the integrated GSM modem. The entire process remained stable, demonstrating that the device is capable of continuous condition monitoring without data loss or communication interruptions.

The primary use case evaluated was condition monitoring of a small-scale diesel engine. The device was mounted on an operating engine to measure:

1. Surface temperature of components using the MLX90614 sensor,
2. Vibrations of the engine block via the GY-521 accelerometer,
3. Sound intensity using the MAX4466 microphone sensor,
4. Rotational speed using an infrared sensor and,
5. Precise time recording through the RTC DS3231 module.

This scenario demonstrates the application of the device in predictive maintenance research, where multi-sensor data are required to detect early signs of engine faults, such as abnormal temperature, unusual noise patterns, performance deviations, or mechanical imbalance.

The NeuraEngineDx system employs a Threshold Logic methodology to determine the operational condition of small-scale fishing vessel engines. Engine condition assessment is performed by comparing measured sensor parameters against predefined thresholds derived from the engine’s operational characteristics. In this study, engine conditions are classified into three categories: Normal, Medium, and Critical. Normal conditions are defined by a temperature range of 60–90°C and noise levels up to 95  dB. Medium conditions are indicated by temperatures of 90–100°C and/or rapid temperature increases within a short period. Critical conditions correspond to temperatures above 100°C or noise levels exceeding 110  dB, signalling potential thermal or mechanical failure in the valve system. Threshold-based was chosen for this initial phase to enable real-time operation on low-power hardware without requiring high-performance processors or costly cloud computing resources.

To evaluate the monitoring capability of NeuraEngineDx, a series of experimental tests were conducted under controlled no-load engine operating conditions with intentionally induced fault scenarios. During testing, the engine was operated at variable rotational speeds within its normal operating range, while no external mechanical load was applied. This approach was adopted to assess the system’s ability to track internal thermal, acoustic, and vibrational dynamics independently of load-induced effects.

**Fig. 11:**
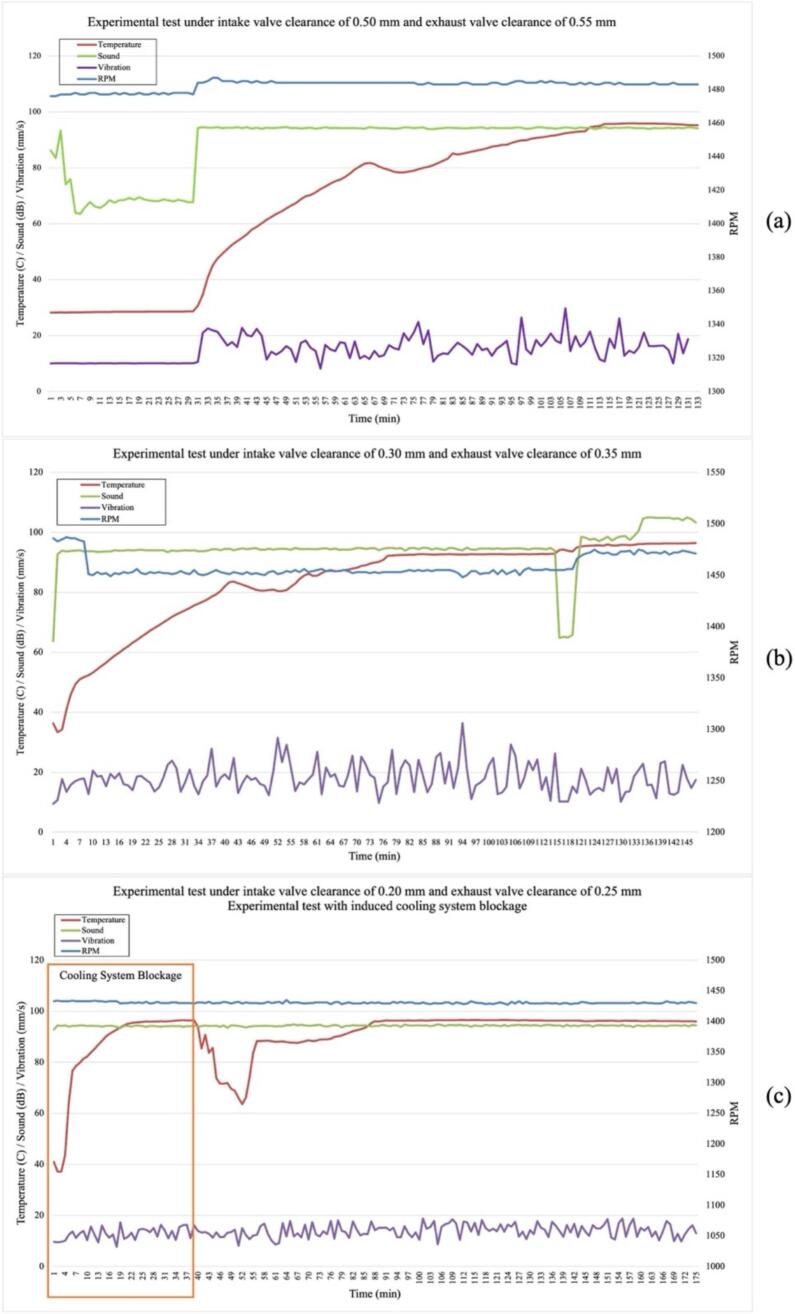
Experimental results under different valve clearance conditions (intake/exhaust): (a) 0.50/0.55 mm, (b) 0.30/0.35 mm, and (c) 0.20/0.25 mm with induced cooling system blockage.

The experimental evaluation consisted of three valve clearance configurations as well as a simulated cooling system failure to assess the fault detection capability of the proposed system. As illustrated in Fig. 11(c), when a coolant flow restriction was intentionally introduced, the MLX90614 infrared temperature sensor recorded a rapid and pronounced increase in engine surface temperature, demonstrating the system’s high sensitivity to thermal anomalies that, if left undetected, could lead to severe internal engine damage. In addition, variations in valve clearance ranging from 0.50/0.55 mm to 0.20/0.25 mm produced measurable changes in engine parameters. These valve clearance changes altered valve timing and increased the mechanical impact intensity between the rocker arm and the valve, which was reflected in distinct trends in temperature, sound level and vibration intensity captured by NeuraEngineDx. The results presented in Fig. 11(a–c) confirm the system’s ability to distinguish different valve clearance conditions in real time, providing a reliable basis for early diagnosis of valve mechanism abnormalities.

Performance characterization was conducted across a range of operational parameters, including temperature variation, engine vibration levels, and sound noise. The results indicate that each sensor responded consistently to changes in engine operating conditions, and the device maintained stable operation throughout the testing period.

**Fig. 12:**
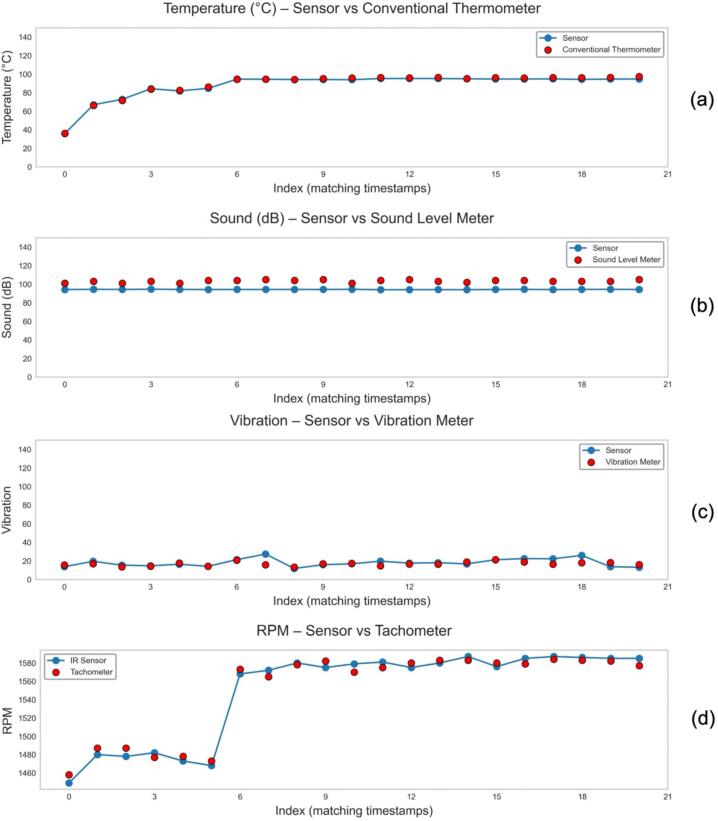
Validation and characterization results of the (a) temperature sensor, (b) sound sensor, (c) vibration sensor and (d) rpm sensor, compared with reference instruments.

The x-axis index in Fig. 12 represents the matched sampling timestamps during the validation stage. Since the measurements were conducted sequentially using different reference instruments, a time gap of several seconds exists between individual data acquisitions. The use of a numerical index allows the synchronization of measurements obtained from the NeuraEngineDx system with those from the manual reference instruments at corresponding measurement events, thereby ensuring accurate visual comparison.

A total of 21 paired measurements were collected for each parameter across the defined operational range. This sample size is statistically adequate for regression-based sensor agreement analysis under controlled laboratory conditions, providing 19 degrees of freedom for correlation estimation. Given that the objective is metrological validation rather than population-level inference, repeated measurements spanning the full operating envelope are more critical than large sample populations.

The temperature measurement results demonstrate a consistent agreement between the sensor and the reference thermometer. As shown in Fig. 12 (a), the recorded temperature increased progressively from approximately 50°C at the initial measurement index to a stable value of around 95°C. No significant deviation was observed, and the two curves almost completely overlapped.

The sound level measurements obtained from the proposed sensor closely matched those of the reference sound level meter. As illustrated in Fig. 12 (b), the recorded sound level remained approximately constant at ∼ 100 dB across all measurement indices. Minor discrepancies between the sensor and the reference instrument were observed; However, these variations did not alter the general measurement pattern or the consistency of the recorded sound levels.

The vibration measurement results exhibit a consistent fluctuating trend, with vibration velocity values ranging between 12 mm/s and 25 mm/s. As illustrated in Fig. 12 (c), the output of the proposed sensor generally follows the response of the reference vibration meter across the entire measurement series. Although minor discrepancies are observed at several measurement points, these differences do not affect the overall trend consistency. This indicates that the sensor is capable of reliably capturing vibration behavior and tracking dynamic variations in vibration velocity, supporting its applicability for vibration monitoring applications.

Validation of the engine rotational speed (RPM) parameter is presented in Fig. 12(d). Measurements were conducted by comparing the digital signal from the infrared (IR) sensor integrated into the NeuraEngineDx system with readings from a standard digital tachometer. The results demonstrate that the system can accurately track engine speed fluctuations, with the average deviation between the sensor and reference instrument remaining within acceptable tolerance limits for monitoring small-scale fishing vessel engines.

**Fig. 13:**
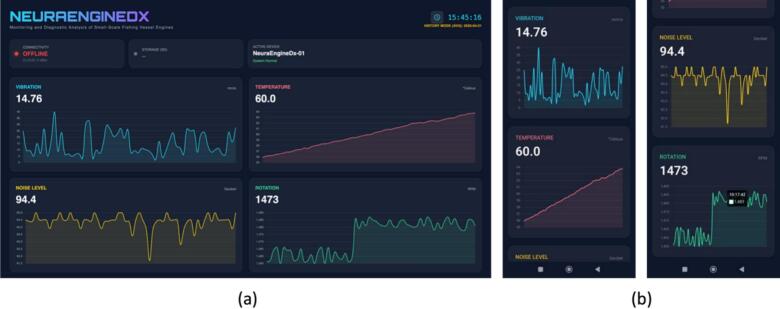
NeuraEngineDx monitoring dashboard during experimental testing: (a) desktop view and (b) smartphone view.

The NeuraEngineDx monitoring dashboard is designed to deliver intuitive and comprehensive information to users. As illustrated in Fig. 13, the dashboard interface provides real-time graphical visualizations and numerical readings for vibration (mm/s), temperature (°C), sound level (dB), and rotational speed (RPM). The interface also incorporates indicators for system connectivity and microSD card storage status. During the experimental evaluation, the dashboard was accessed via mobile devices or laptops connected to the internet network supplied by portable modem, enabling continuous monitoring of engine operating conditions without requiring operators to remain in close proximity to the running engine.

**Fig. 14:**
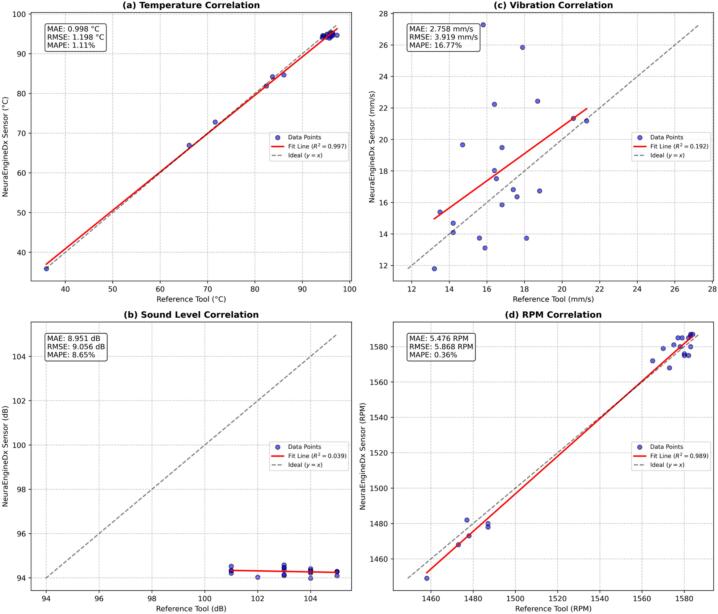
Correlation analysis and accuracy validation of NeuraEngineDx sensors against industry-standard reference instruments. The plots illustrate the correlation performance and statistical error metrics for: (a) Temperature (°C), (b) Sound level (dB), (c) Vibration (mm/s), dan (d) RPM.

To further evaluate the accuracy and precision of the system across its operational range, correlation plots and comprehensive error metrics were constructed, as shown in Fig. 14. The performance of the NeuraEngineDx system was assessed using absolute error metrics, including MAE, RMSE, and MAPE, along with correlation analysis (R2). While the system demonstrates high precision in monitoring temperature and RPM, lower R2 values were observed for the acoustic and vibration parameters, despite their relatively low absolute error values. Based on Table 8, the system achieved R2 values of 0.997 and 0.989, respectively, indicating an almost perfect linear correlation with the reference instruments. These results confirm the system’s strong capability to accurately track dynamic changes in these parameters during engine testing. In addition, the low MAE value for temperature measurements (0.998°C) further validates the accuracy of the sensing approach employed.

**Table 8:** Accuracy metrics of NeuraEngineDx system for engine monitoring.

**Parameter**	**MAE**	**RMSE**	**MAPE**	**R^2^**
Temperature	0.998°C	1.198°C	1.11%	0.997
RPM	5.476 RPM	5.868 RPM	0.36%	0.989
Sound	8.951 dB	9.056 dB	8.65%	0.039
Vibration	2.758 mm/s	3.919 mm/s	16.77%	0.192

The lower R^2^ values observed for the sound (0.039) and vibration (0.192) sensors are attributed to several factors affecting measurement fidelity. For acoustic measurements, the reference instrument recorded fluctuations between 101–105  dB, whereas the sensor output remained near 94  dB. Although the MAE of 8.951  dB remains within a tolerable range, the limited sensor sensitivity resulted in an R^2^ value close to zero, highlighting the need for improved sensor responsiveness to capture subtle variations at higher intensity levels. In the case of vibration monitoring, the high scatter of measurements relative to a small measurement range (12–22  mm/s) caused significant variance. The R^2^ value of 0.192 reflects this noise, while the system maintained a reasonable absolute accuracy with an MAE of 2.758  mm/s.

To complement the accuracy assessment (MAE, RMSE, and MAPE), uncertainty analysis was performed following the Guide to the Expression of Uncertainty in Measurement framework to quantify the precision of the system. Both random fluctuations and sensor specifications were considered to calculate the expanded uncertainty at a 95% confidence level. Table 9 summarizes the comprehensive uncertainty analysis for the four primary parameters measured during engine testing.

**Table 9:** Expanded uncertainty analysis (k = 2, 95% confidence level).

**Parameter**	**Unit**	**n**	**S**	**u_A_**	**u_B_**	**u_C_**	**U**	**Rel. U (%)**
Temperature	°C	21	0,97	0,21	0,29	0,36	0,72	0,81
Sound	dB	21	1,41	0,31	1,15	1,2	2,39	2,32
Vibration	mm/s	21	3,79	0,83	0,29	0,88	1,75	10,51
Rpm	RPM	21	6,01	1,31	2,89	3,17	6,34	0,41

The results indicate high stability for temperature and RPM measurements, with low relative uncertainties of 0.81% and 0.41%, respectively, confirming excellent repeatability and suitability for real-time monitoring. Noise level measurements exhibited moderate relative uncertainty (2.32%), primarily due to environmental variability and analog signal fluctuations, but remained acceptable for condition monitoring applications. Vibration measurements showed the highest relative uncertainty (10.51%), likely due to the dynamic nature of mechanical vibrations and the sensitivity of MEMS sensors to mounting conditions.

### 7.3 Hardware capabilities

NeuraEngineDx was developed as a monitoring system for small-scale fishing vessel engines, providing technical capabilities to support fishers in assessing engine conditions and making informed predictive maintenance decisions. The device is able to operate multiple sensors simultaneously, including modules for temperature, vibration, noise level, and rotational speed. Temperature and rotational measurements achieve high accuracy, supported by the MLX90614 sensor with a tolerance of ± 0.5°C within its operational range. The system provides continuous data logging to a MicroSD card without packet loss and maintains stable internet connectivity for real-time data transmission to a cloud server. Its enclosure, constructed from Nylon Carbon, offers durability in harsh field conditions and withstands temperatures up to 180°C. A built-in TFT display enables immediate on-site monitoring, while precise time synchronization is ensured by the RTC DS3231 module and NTP server.

NeuraEngineDx demonstrates monitoring of primary engine indicators, achieving R^2^ values of 0.997 for temperature and 0.989 for rotational speed, thereby supporting the early detection of thermal and mechanical anomalies. Its multi-sensor capability enables real-time identification of abnormal conditions, such as cooling system blockages and valve clearance deviations, through integrated analysis of vibration, acoustic, and thermal trends. Efficient power management is provided by a 9000  mAh battery, allowing continuous operation for up to 34  h, exceeding the typical duration of a one-day fishing trip. The system offers dual-interface monitoring, with local visualization via the TFT LCD for offline operation and a web-based dashboard for remote data analysis when internet connectivity is available.

### 7.4 Limitation and future Works

The NeuraEngineDx system demonstrates promising performance in small-scale engine monitoring; however, several limitations remain that warrant further investigation.

First, The MAX4466 acoustic sensor employs a time-domain estimation of sound pressure level to enable real-time processing on the ESP32 microcontroller. While effective for capturing engine noise, this approach has limited ability to distinguish ambient maritime noise from engine-specific acoustic components. To mitigate this effect, an Exponential Moving Average filter is implemented in the firmware. Nevertheless, more advanced frequency-domain processing or sensor fusion techniques are recommended for future work to enhance system robustness against environmental noise interference.

Second, the lower correlation observed in vibration data highlights the challenge of filtering mechanical noise from single-cylinder engines using low-cost MEMS sensors. To mitigate these effects, At the signal level, band-pass or adaptive digital filtering can suppress transient spikes and resonance artifacts. Additionally, employing higher-resolution accelerometers with a wider dynamic range may further improve measurement stability and correlation performance.

Third, cloud-based data transmission is highly dependent on the quality of available internet signals in fishing areas. When all sensors operate simultaneously at maximum sampling frequencies, the data rate may decrease, and power consumption increases due to prolonged modem activity, indicating practical constraints typical of compact, field-deployed IoT systems.

To advance the system toward full industrial readiness, several directions for future work are proposed:

a. Enhancing the sensitivity of vibration and acoustic modules is a primary goal to detect small-magnitude fluctuations more precisely, thereby improving correlation (R^2^) with reference instruments.
b. The system requires rigorous testing under harsh operational conditions, including:
  • Salt Spray Test: To evaluate the corrosion resistance of sensor materials and casing.
  • High-Temperature Engine Room Test: To assess the thermal stability of electronic circuits during continuous operation in elevated ambient temperatures.
  • Waterproof Test: To ensure compliance with IP standards, protecting the system from moisture and splashes during engine operation.
c. While the current implementation of NeuraEngineDx is primarily focused on condition monitoring, the system’s capability to collect real-time, multi-parameter data establishes a robust foundation for the integration of artificial intelligence (AI) in future developments. The datasets generated by the device can be leveraged to train machine learning algorithms capable of performing automatic fault detection and supporting predictive maintenance strategies. By analyzing patterns across vibration, temperature, acoustic, and rotational speed signals, AI-based models have the potential to identify subtle anomalies and anticipate component failures, thereby enhancing the system’s accuracy and operational reliability in small-scale marine engines.
d. Long term testing under real operational conditions on small-scale fishing vessels to assess reliability in complex and dynamic maritime environments.
e. Validation across different engine loads to establish operational robustness and sensor accuracy under variable power conditions.

Overall, while NeuraEngineDx demonstrates capabilities in monitoring small-scale marine engines, further work is required to validate its performance under diverse operational conditions, enhance sensor robustness, and explore advanced predictive approaches.

## Ethics statements

This work does not involve the use of any human or animal subjects.

## CRediT authorship contribution statement

**Yuniar Endri Priharanto:** Writing – original draft, Methodology, Conceptualization. **Indra Jaya:** Writing – review & editing, Validation, Supervision, Methodology. **Ayi Rahmat:** Writing – review & editing, Software. **Medria Kusuma Dewi Hardhienata:** Writing – review & editing, Methodology, Formal analysis, Conceptualization. **Donwill Panggabean:** Writing – review & editing, Visualization.

## Declaration of competing interest

The authors declare that they have no known competing financial interests or personal relationships that could have appeared to influence the work reported in this paper.
