# Supplementary material for: NeuraEngineDx: An IoT condition monitoring device for Small-Scale fishing vessel engines
Source: HardwareX. 2026 Mar 21;26:e00763. doi: 10.1016/j.ohx.2026.e00763 (PMC13051961; doi:10.1016/j.ohx.2026.e00763)
Supplement: Supplementary Data 1 [file mmc1.pdf]

Appendix A. Manual PCB Fabrication via Chemical Etching

Although professional fabrication is strongly recommended for long-term deployment in maritime environments, the following procedure may be used for rapid field prototyping. The general fabrication steps include:

1. Layout Printing.
   Print the PCB trace design onto transfer paper using a laser printer with mirrored orientation.
2. Trace Transfer.
   Attach the printed design onto the copper-clad board using a hot iron (180–200°C) for approximately 5 minutes until the toner is fully transferred.
3. Etching Process.
   Immerse the board into a ferric chloride ($FeCl_3$) solution until the exposed copper areas are completely dissolved, typically 10–15 minutes.
4. Cleaning and Coating.
   Remove residual toner using acetone, then coat the copper traces with molten solder or solder flux to prevent oxidation.
5. Drilling Holes.
   Drill component and connector holes using a mini drill with a diameter of 0.8–1.0 mm.
6. Final Coating/Masking (Optional).
   Apply a protective film or masking paint on the surface to enhance moisture and corrosion resistance.

Manual assembly is recommended for the single-board version. If creating vias is not feasible, the two layers can be connected using a thin jumper wire (0.3 mm).

Appendix B. Detailed Firmware Troubleshooting

The following table details error codes and corresponding technical solutions for developers.

**Problem**	**Common Cause**	**Recommended Solution**
ESP32 not detected	Faulty data cable or missing CP210x driver	Use a high-quality data cable; install the appropriate USB-to-UART (CP210x) driver.
“SD Card Mount Failed”	Incompatible SD card format or loose SPI connection	Format the SD card to FAT32; inspect and resolder the SD module SPI connections if necessary.
WiFi connection failure	Weak signal strength or incorrect credentials	Use a 2.4 GHz hotspot; verify the SSID and password configuration.
Frozen sensor readings	I2C bus communication interference	Inspect SDA and SCL wiring; add appropriate pull-up resistors if cable length is excessive.
